# Supplementary material for: Adherence to Child Feeding Practices and Child Growth: A Retrospective Cohort Analysis in Cambodia
Source: Nutrients. 2020 Dec 31;13(1):137. doi: 10.3390/nu13010137 (PMC7823716; doi:10.3390/nu13010137)
Supplement: Supplementary file 1 [file nutrients-13-00137-s001.pdf]

Supplementary Table S1: The relationship of adherence to age-appropriate feeding practices and other determinants with child Height-for-Age (HAZ).

					**HAZ**				
	**Model 1**[a]			**Model 2**[b]			**Model 3**[c]		
Full adjusted model	***Total number of adherence to MDD or MMF*** (n=1,918 ; observations=8,873)			***Total number of adherence to MAD*** (n=1,918 ; observations=8,873)			***Total number of adherence to ADF*** (n=1,918 ; observations=8,873)		
**Fixed effects**	Cof	95CI %	***p-Value***	Cof	95CI %	***p-Value***	Cof	95CI %	***p-Value***
Sex (boy)	-0.10	-0.16 - -0.05	***<0.001***	-0.12	-0.16 - -0.05	***<0.001***	-0.11	-0.16 - -0.05	***<0.001***
Age (in month)	-0.10	-0.10 - -0.09	***<0.001***	-0.10	-0.10 - -0.09	***<0.001***	-0.10	-0.10 - -0.09	***<0.001***
Age^2 (in month)	0.002	0.0015 - 0.0019	***<0.001***	0.002	0.0015 - 0.0019	***<0.001***	0.002	0.0015 - 0.0018	***<0.001***
Total number of adherence to *MDD (Never)*									
1 or 2 times	0.09	0.02 - 0.17	***0.010***						
3 or always	0.10	-0.01 - 0.20	***0.070***						
Total number of adherence to *MMF(Never)*									
1 or 2 times	-0.16	-0.30 - -0.01	***0.031***						
3 or always	-0.30	-0.45 - -0.15	***<0.001***						
Total number of adherence *to MAD (Never)*									
1 or 2 times				*0.01*	*-0.05 - 0.08*	***0.710***			
3 or always				-0.09	-0.20 - 0.02	***0.095***			
Total number of adherence *to ADF(Never)*									
1 or 2 times							-0.001	-0.07 - 0.07	***0.985***
3 or always							0.38	0.26 - 0.50	***<0.001***
Maternal education (years)	0.04	0.03 - 0.04	***<0.001***	0.03	0.03 - 0.04	***<0.001***	0.03	0.02 - 0.04	***<0.001***
Wealth index	0.10	0.07 - 0.12	***<0.001***	0.10	0.07 - 0.12	***<0.001***	0.10	0.08 - 0.12	***<0.001***
Caretaker drinking water was treated	0.09	0.01 - 0.16	***0.023***	0.09	0.02 - 0.12	***0.016***	0.08	0.01 - 0.16	***<0.001***
Improved toilet	0.13	0.06 - 0.19	***<0.001***	0.14	0.07- 0.21	***<0.001***	0.14	0.07 - 0.21	***<0.001***
**Random effect**									
Variances of random intercept	0.04	0.01 - 0.193		0.04	0.01 - 0.21		0.04	0.008 - 0.21	
Variance of random slope	0.003	0.002 - 0.003		0.003	0.002 - 0 .003		0.003	0.002 - 0.003	
Variance of residuals	0.62	0.60 - 0.64		0.62	0.60 - 0.64		0.62	0.60 - 0.64	
Log-likelihood		-12183.22			-12195.77			-12175.54	
LR test				***<0.001***			***<0.001***		***<0.001***

[a] Model 1 presents linear mixed-effects model of the relationship between adherence to MDD or MMF and other determinants whit HAZ.

[b] Model 2 presents linear mixed-effects model of the relationship between adherence to MAD and other determinants with HAZ.

[c] Model 3 presents linear mixed-effects model of the relationship between adherence of ADF and other determinants with HAZ.

MDD= minimum dietary diversity, MMF= minimum meal frequency; MAD=minimum acceptable diet; and ADF=age-appropriate daily feeding.

Supplementary Table S2: The relationship of adherence to age-appropriate feeding practices and other determinants with child Weight-for-Height (WHZ).

WHZ									
	**Model 1[a]**			**Model 2[b]**			**Model 3[c]**		
Full adjusted model	***Total number of adherence to MDD or MMF*** (n=1,995; observations=9,231)			***Total number of adherence to MDA*** (n=1,995; observations= 9,231)			***Total number of adherence to ADF*** (n=1,995; observations= 9,231)		
Fixed effects	Cof	95CI %	*p-Value*	Cof	95CI %	*p-Value*	Cof	95CI %	*p-Value*
Sex (boy)	-0.06	-0.11 - -0.01	***0.020***	-0.06	-0.11 - -0.01	***0.015***	-0.06	-0.11 - -0.01	***0.018***
Age (in month)	-0.05	-0.05 - -0.04	***<0.001***	-0.05	-0.05 - -0.04	***<0.001***	-0.05	-0.06 - -0.04	***<0.001***
Age^2 (in months)	0.001	0.0009 - 0.0012	***<0.001***	0.001	0.0008 - 0.001	***<0.001***	0.001	0.009 - 0.001	***<0.001***
Total number of adherence *to MDD (Never)*									
1 or 2 times	0.13	0.07 - 0.19	***<0.001***						
3 or always	0.23	0.13 - 0.32	***<0.001***						
Total number of adherence *to MMF(Never)*									
1 or 2 times	0.04	-0.09 - 0.16	***0.568***						
3 or always	-0.03	-0.16 - 0.10	***0.636***						
Total number of adherence *to MAD (Never)*									
1 or 2 times				0.10	0.05 - 0.16	***<0.001***			
3 or always				0.17	0.08 - 0.27	***<0.001***			
Total number of adherence *to ADF (Never)*									
1 or 2 times							0.02	-0.04 - 0.07	***0.613***
3 or always							0.03	-0.08 - .14	***0.609***
Maternal education (years)	0.01	0.001 -0.02	***0.030***	0.01	0.001 - 0.02	***0.022***	0.009	0.001 - 0.02	***0.019***
Wealth index	0.09	0.07 - 0.11	***<0.001***	0.09	0.07 - 0.11	***<0.001***	0.09	0.07- 0.11	***<0.001***
**Random effects**									
Variances of random intercept	0.01	0.001 - 0.04		0.01	0.001 - 0.04		0.01	0.001 - 0.04	
Variance of random slope	0.002	0.001 - 0.002		0.002	0.001 - 0 .002		0.002	0.001 - 0.002	
Variance of residuals	0.59	0.57 - 0.61		0.587	0.57 - 0.61		0.59	0.57 - 0.61	
Log-likelihood		-12075.77			-12080.43			-12089.34	
LR test			***<0.001***			***<0.001***			***<0.001***

[a] Model 1 presents linear mixed-effects model of the relationship between adherence to MDD or MMF and other determinants with WHZ.

[b] Model 2 presents linear mixed-effects model of the relationship between adherence to MAD and other determinants with WHZ.

[c] Model 3 presents linear mixed-effects model of the relationship between adherence to ADF and other determinants with WHZ.

MDD= minimum dietary diversity, MMF= minimum meal frequency; MAD=minimum acceptable diet; and ADF=age-appropriate daily feeding.

Supplementary Table S3: The relationship of adherence to age-appropriate feeding practices and other determinants with child concurrence wasting and stunting (WaSt*)*.

	WaSt								
	**Model 1[a]**			**Model 2[b]**			**Model 3[c]**		
**Full adjusted model**	***Total number of adherence to MDD or MMF* (n=1,995;observations=9,231)**			***Total number of adherence to MAD* (n=1,995; observations=9,231)**			***Total number of adherence to ADF* (n=1,995; observations=9,231)**		
**Fixed effect**	AOR	95CI %	*p-Value*	AOR	95CI %	*p-Value*	AOR	95CI %	*p-Value*
Age (in month)	1.40	1.30 - 1.50	***<0.001***	1.40	1.30 - 1.50	***<0.001***	1.39	1.29 - 1.50	***<0.001***
Age^2 (in months)	0.992	0.991 - 0.995	***<0.001***	0.993	0.991 - 0.994	***<0.001***	0.992	0.990 - .994	***<0.001***
Total number of adherence to *MDD (Never)*									
1 or 2 times	0.71	0.37- 1.34	***0.286***						
3 or always	0.74	0.26 - 2.05	***0.558***						
Total number of adherence *to MMF(Never)*									
1 or 2 times	0.54	0.14 - 2.06	***0.370***						
3 or always	0.97	0.25 - 3.73	***0.969***						
Total number of adherence to *MAD (Never)*									
1 or 2 times				0.85	0.47 - 1.56	***0.619***			
3 or always				1.23	0.43 - 3.50	***0.703***			
Total number of adherence *to ADF(Never)*									
1 or 2 times							1.27	0.68 - 2.37	0.451
3 or always							0.45	0.09 - 2.30	0.338
Maternal education (years)	0.83	0.76 - 0.90	***<0.001***	0.82	0.76 - 0.90	***<0.001***	0.83	0.76 - 0.90	***<0.001***
Wealth index	0.59	0.46 - 0.74	***<0.001***	0.59	0.46 - 0.74	***<0.001***	0.58	0.46 - 0.73	***<0.001***
**Random effect**									
Variances of random intercept	10.20	7.55 - 13.78		10.26	7.59 - 13.87		10.17	7.53 - 13.74	
Log-likelihood		-1192.66			-1194.69			-1193.81	
LR test			***<0.001***			***<0.001***			***<0.001***

[a] Model 1 presents mixed-effects logistic regression of the relationship between adherence to MDD or MMF and other determinants with WaSt.

[b] Model 2 presents mixed-effects logistic regression of the relationship betweenadherence to MAD and other determinants with WaSt.

[c] Model 3 presents mixed-effects logistic regression of the relationship betweenadherence to ADF and other determinants with WaSt.

MDD= minimum dietary diversity, MMF= minimum meal frequency, MAD=minimum acceptable diet; ADF=age-appropriate daily feeding.
